# Trade-offs drive resource specialization and the gradual establishment of ecotypes

**DOI:** 10.1186/1471-2148-14-113

**Published:** 2014-05-29

**Authors:** Bjørn Østman, Randall Lin, Christoph Adami

**Affiliations:** 1 Department of Microbiology and Molecular Genetics, Michigan State University, MI 48824 East Lansing, USA; 2 BEACON Center for the Study of Evolution in Action, Michigan State University, 48824 East Lansing, USA; 3 California Institute of Technology, CA 91125 Pasadena, USA

**Keywords:** Trade-offs, Speciation, Ecotypes, Selection, Adaptive radiation, Simulation, Phyletic evolution

## Abstract

**Background:**

Speciation is driven by many different factors. Among those are trade-offs between different ways an organism utilizes resources, and these trade-offs can constrain the manner in which selection can optimize traits. Limited migration among allopatric populations and species interactions can also drive speciation, but here we ask if trade-offs alone are sufficient to drive speciation in the absence of other factors.

**Results:**

We present a model to study the effects of trade-offs on specialization and adaptive radiation in asexual organisms based solely on competition for limiting resources, where trade-offs are stronger the greater an organism’s ability to utilize resources. In this model resources are perfectly substitutable, and fitness is derived from the consumption of these resources. The model contains no spatial parameters, and is therefore strictly sympatric. We quantify the degree of specialization by the number of ecotypes evolved and the niche breadth of the population, and observe that these are sensitive to resource influx and trade-offs. Resource influx has a strong effect on the degree of specialization, with a clear transition between minimal diversification at high influx and multiple species evolving at low resource influx. At low resource influx the degree of specialization further depends on the strength of the trade-offs, with more ecotypes evolving the stronger trade-offs are. The specialized organisms persist through negative frequency-dependent selection. In addition, by analyzing one of the evolutionary radiations in greater detail we demonstrate that a single mutation alone is not enough to establish a new ecotype, even though phylogenetic reconstruction identifies that mutation as the branching point. Instead, it takes a series of additional mutations to ensure the stable coexistence of the new ecotype in the background of the existing ones.

**Conclusions:**

Trade-offs are sufficient to drive the evolution of specialization in sympatric asexual populations. Without trade-offs to restrain traits, generalists evolve and diversity decreases. The observation that several mutations are required to complete speciation, even when a single mutation creates the new species, highlights the gradual nature of speciation and the importance of phyletic evolution.

## Background

Trade-offs present limitations to the adaptive potential of organisms and are commonly thought of as the reason why we observe such an abundance of species, rather than just a few that have adapted to any conditions that life may present [1]. While the divergence of allopatric species is sustained by geographic barriers, sympatric populations can split through the action of negative frequency-dependent selection, in which rare types gain a fitness advantage over types of high frequency, for example by tapping into an unused and abundant resource. This force for diversification can be driven by fitness trade-offs between different niches, without which a single generalist phenotype could sweep the population. Empirically two- and three-way trade-offs have been observed in several microbial systems, such as maximum growth rate vs. yield (i.e., biomass produced per unit resource) in *E. coli*[2], nitrogen and phosphorous affinity and cell volume in phytoplankton [3], or ability to adapt to a CO _2_ enriched environment and competitive ability [4]. Modeling approaches have explored the trade-offs between two traits, such as rate of resource acquisition vs. biomass production (rate vs. yield) and maximum uptake rate vs. affinity [5,6], leading to co-existence of two ecotypes on one resource.

To address the influence of trade-offs and other factors on diversification in asexual organisms, we study a model where fitness gained from resources is dependent on explicitly modeled trade-offs between the traits that control resource use. We aim to quantify the impact that trade-offs have on the degree of resource specialization, measured as the number of distinct ecotypes that can co-exist. Fitness is given as a function of the organism’s ability to utilize the available resources modeled on the Monod equation [7], modified such that having high affinity for more resources comes at a cost in fitness leading to trade-offs. Spatial structure is absent from the model, making the system strictly sympatric, as opposed to weakly sympatric, which would retain a spatial component that can affect the dynamics of the system (e.g., [8-10]).

We restrict our model to asexual organisms, showing how resource specialization leads to adaptive radiation in the absence of reproductive isolation. This best mirrors evolutionary and ecological dynamics of unicellular, asexual organisms in which mating and genetic recombination are absent. Such organisms include bacteria, in which adaptive radiation has been observed on several occasions [11-13], and plankton, which forms the basis for the question of how several species occupying the same niche can coexist seemingly indefinitely, the so-called *paradox of the plankton*[14-21].

We determine the number of species using the *Ecological Species Concept*[22-24], which defines a species as “a lineage which occupies an adaptive zone minimally different from that of any other lineage in its range and which evolves separately from all other lineages outside its range,” without claiming that this definition is always appropriate. Species by this definition are also denoted *ecotypes*, and here we use the two terms interchangeably.

Depending on the parameters governing resource abundance, mutational effects, and trade-offs leading to negative frequency-dependent selection, we observe resource competition giving rise to either generalists or specialists. By tracking the evolving organisms and reconstructing the phylogenetic relationship of the surviving lineages we can identify the exact mutations that cause the initial divergence between lineages. This analysis then enables us to distinguish between anagenetic and cladogenetic change, addressing a long-standing question about whether macroevolutionary change proceeds by gradualistic or punctuated modes (e.g. [25]).

## Results

**Figure 1:**
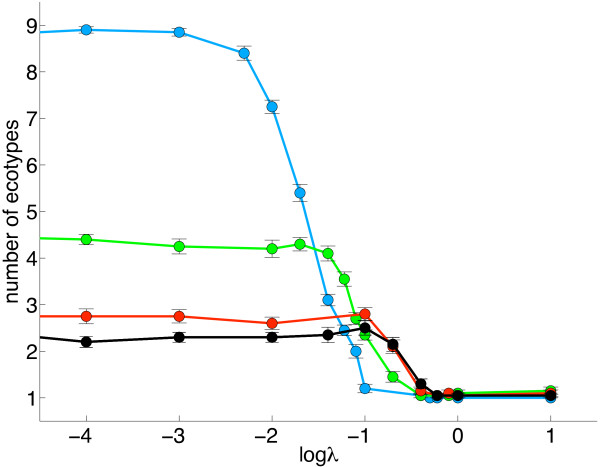
**Number of ecotypes as a function of resource influx for different levels of trade-off.** A low influx creates a pressure for the population to split into different ecotypes because it this enables selection to differentiate between phenotypes that use different resources at different abundances. The degree of specialization does not diminish when the influx decreases (see Discussion). More specialists evolve when trade-offs are more severe, essentially creating one niche per resource for the highest cost. Even when having a high resource affinity is not penalized, the population still fragments into on average two stable lineages. Blue markers: (*σ*_1_,*σ*_2_) = (10,1), green: (*σ*_1_,*σ*_2_) = (1,0.1), red: (*σ*_1_,*σ*_2_) = (0.1,0.01), black: (*σ*_1_,*σ*_2_) = (0,0). *N*=1000, *μ*=0.05. Each datum is the average of 20 simulations and error bars are s.e.m.

We carried out simulations with different values of resource influx, *λ*, cost-parameters, *σ*_1_ and *σ*_2_, population size, *N*, and mutation rate, *μ*, and examined their impact on the degree of specialization (see Table 1 for a list of model parameters and their values). By far the two biggest factors affecting the degree of specialization are the resource influx and trade-offs (Figure 1). The number of ecotypes *n*_*t*_ is first and foremost a function of the amount of resources that flow into the system. When this rate is high, the number of ecotypes is close to one, and when it is low, the number of types increases. Contrary to results from experiments with bacteria [13] and digital evolution [26], which both found unimodal distributions of diversity as a function of resource influx, here the number of types does not decrease even for very low influx (see Discussion). When the influx is low, the number of ecotypes is strongly dependent on the cost-parameters, with high cost leading to more ecotypes. As the influx increases there is a transition where the number of ecotypes drops to one. When the resource influx is high, generalists dominate, and adaptive radiation and specialization do not occur. The dynamics of the model depend on the resource abundance, such that selection can only differentiate between phenotypes using different resources when those resource differences result in fitness differences that are larger than the inverse of the population size, *s*≥1/*N* (e.g., [27]). If the fitness difference between two organisms is smaller than this value, then genetic drift has a larger influence on the dynamics, and selection will not favor either organism. In that case, an incentive for the population to split into different groups ceases to exist, and generalists evolve. The more severe the cost is, the more specialists evolve, essentially creating one niche per resource for the highest cost. Even when there is no cost of having high resource affinity, the population still fragments into approximately two stable lineages, but this is an effect of fixation by drift being a very slow process. When the zero-mutation rate experiments are run for a much longer time, the most dominant phenotype eventually replaces all other leaving just one ecotype. This expectation also forms the basis of the *paradox of the plankton*, where many species of plankton coexist on seemingly very few distinct resources [14].

**Table 1:** Model parameters and values used in the simulations

*N*	Population size	100, 1000, 5000
*R*	Number of resources and traits (resource affinities)	9
*c*_*i* *k*_	Resource affinity of an individual I for resource k	[0, 1]
*μ*	Per-trait mutation rate	0.01, 0.05
*γ*_*k*_	Half-saturation of resource k	100
*σ*_1_	Cost of having high resource affinity	0, 0.1, 1, 10
*σ*_2_	Cost of having many non-zero resource affinities	0, 0.01, 0.1, 1
*λ*_*k*_	Influx of resource k per individual	[ 10^-4^, 10]
*c*_0_	Resource decay rate	0.1
*r*_*k*_	Abundance of resource k	[ *Nλ*_*k*_, *Nλ*_*k*_/*c*_0_]

**Figure 2:**
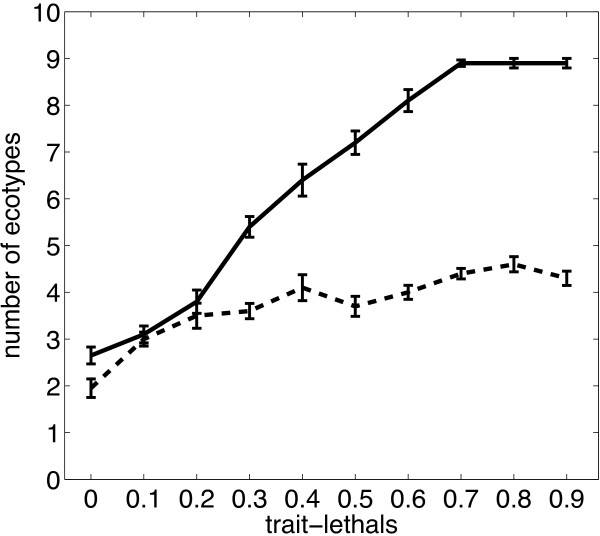
**Increasing the fraction of trait-lethals increases the degree of specialization.** Solid line: (*σ*_1_,*σ*_2_) = (10, 1), dashed line: (*σ*_1_,*σ*_2_) = (1, 0.1). Each datum is the mean of 10 simulations ± std. dev., except for zero and 70*%* trait-lethals, which are for 20 simulations. log *λ*=-4, *N*=1000, *μ*=0.05. Error bars are s.e.m.

Trait-lethal mutations have a large impact on the degree of specialization. Without the possibility of traits mutating to zero, niche breadth is nearly always at the maximum of one and *n*_*t*_ is severely depressed compared to simulations that have 70% trait-lethals (Table 2). The number of ecotypes is an increasing function of the fraction of trait-lethals (Figure 2), and has a strong enhancing effect when trade-offs are strong. In the case of less strong trade-offs there is a pronounced difference between zero and 10% trait-lethals.

**Table 2:** The effect of trait-lethal mutations is very strong

**log** ***λ***	***σ***_**1**_	***σ***_**2**_	**〈** ***n***_***t***_**〉**^***a***^	***B***^***a***^	**〈** ***n***_***t***_**〉**^***b***^	***B***^***b***^
-1	1	0.1	1.35±0.49	1.00	2.35±0.49	0.45
-2	1	0.1	2.05±0.76	0.99	4.20±0.83	0.37
-3	1	0.1	2.25±0.85	0.98	4.25±0.72	0.36
-4	1	0.1	1.95±0.89	0.99	4.40±0.50	0.36
-1	10	1	1.05±0.22	0.81	1.20±0.41	0.11
-2	10	1	1.95±0.76	0.91	7.25±0.64	0.13
-3	10	1	2.50±0.83	0.91	8.85±0.37	0.12
-4	10	1	2.65±0.81	0.89	8.90±0.31	0.12

The degree of specialization is much lower and niche breadth is much higher when mutation can only increase or decrease resource affinity by 0.1. ***〈*** *n*_*t*_ ***〉*** is the mean of 20 simulations ***±*** std. dev. *N* ***=*** 1000, ***μ=*** 0.05. ^*a*^Without trait-lethals. ^*b*^With trait-lethals.

We estimated the *most recent common ancestor* (MRCA) by tracing the lines of descent backwards and noting when they coalesce. If a population does not split into more than one stable subpopulation, the MRCA should be relatively recent. When lineages coexist for a long time, the MRCA will be far in the past. In most cases the first split occurs very early in the simulations, i.e., two or more organisms in the starting population start lineages that persist until the end of the runs. We also ran simulations in a neutral fitness landscape, where all organisms have the same fitness, and found that the MRCA is always very close to the present. In this case genetic drift causes the population to descend from a single ancestor not very far in the past, as expected.

The degree of specialization does not change when simulations are run longer. When run for 80,000 updates (see Methods) before turning off mutations, neither *n*_*t*_ nor *B* changes significantly compared to running them for 40,000 updates. What does change in between these two lengths of the simulations is the mean resource affinity, which continues to increase, except in simulations in which high affinities are heavily penalized (e.g., *σ*_1_=10).

The population needs to be of a certain size in order to effectively specialize. For the small population size of *N*=100 the number of ecotypes was consistently much lower than for *N*=1000. None of the parameter sets tried gave an average *n*_*t*_ greater than 2. Increasing the population size to *N*=5000 results in only slightly more ecotypes compared to *N*=1000 (Table 3).

**Table 3:** The population needs to be of a certain size in order for specialists to evolve

**log** ***λ***	***σ***_**1**_	***σ***_**2**_	**〈** ***n***_***t***_**〉**^***a***^	**〈** ***n***_***t***_**〉**^***b***^	**〈** ***n***_***t***_**〉**^***c***^
-2	10	1	8.90±0.31	7.25±0.64	1.00±0.00
-3	0.1	0.01	3.25±1.16	2.75±0.64	1.60±0.50
-3	1	0.1	5.25±1.21	4.25±0.72	1.60±0.50
-3	10	1	8.90±0.31	8.85±0.37	1.40±0.50

A population size of *N* ***=*** 100 is too small to accommodate different ecotypes, while 1000 is large enough that selection can distinguish between different phenotypes and negative frequency-dependent selection is able to sustain diversity. Increasing *N* beyond 1000 has only a small positive effect on the degree of specialization. ***μ=*** 0.05. ^*a*^*N* ***=*** 5000. ^*b*^*N* ***=*** 1000. ^*c*^*N* ***=*** 100.

**Figure 3:**
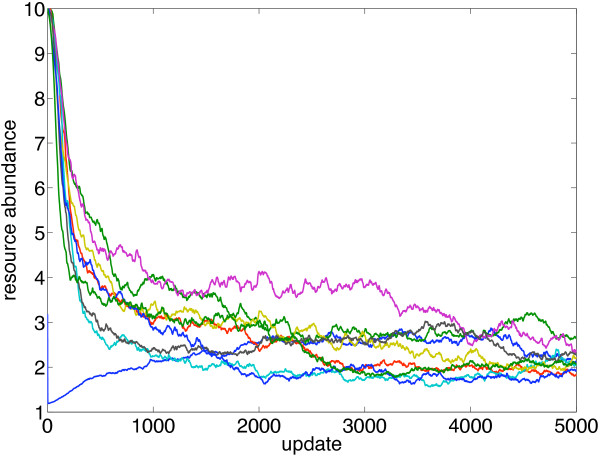
**Resource abundance for nine resources as a function of time.***N*=1000, *μ*=0.05, *λ*=10^-3^, *σ*_1_=1, *σ*_2_=0.1.

Simulations start with a homogeneous population consisting of specialists for one resource (*c*_1_=0.1). However, starting with generalists that have a non-zero affinity for all resources results in the same degree of specialization (Table 4). Resource abundances usually equilibrate within a few hundred updates (Figure 3), and hence the number of ecotypes is unaffected by the resource abundance at the beginning of the simulations.

**Table 4:** A comparison between simulations where the initial homogeneous population consists of specialists or generalists

**log** ***λ***	***μ***	***σ***_**1**_	***σ***_**2**_	**〈** ***n***_***t***_**〉**^***a***^	**〈** ***n***_***t***_**〉**^***b***^
-3	0.05	1	0.1	4.30±0.80	4.25±0.72
-3	0.01	1	0.1	3.00±0.65	3.00±0.73

The number of ecotypes is not different between the two initial conditions. *N* ***=*** 1000. ^*a*^starting with generalists. ^*b*^starting with specialists.

### Gradual establishment of ecotypes

**Figure 4:**
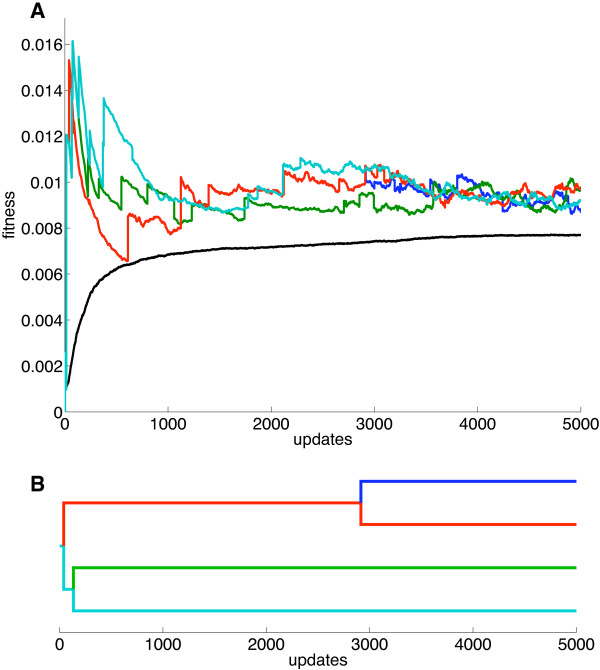
**Simulation resulting in the four ecotypes shown in Table **5.**(A)** Fitness as a function of time for each of the four ecotypes (blue, red, green, cyan) and the population mean fitness (black). **(B)** Phylogram showing the evolutionary relationship between the four ecotypes. *N*=1000, *μ*=0.05, *λ*=10^-3^, *σ*_1_=1, *σ*_2_=0.1, starting with a homogeneous population of specialists with affinity equal to 0.1 for the first resource and zero for the rest.

In order to gain insights into the mechanics of specialization, we reconstructed all mutations on the line of descent for a simulation that resulted in four ecotypes. This enabled us to track the changes that eventually lead to diversification, record how early they arise, and whether they appear gradually or within a short time-span. Because tracking all *N* phenotypes is computationally demanding, we were not able to run simulations longer than 5,000 updates when reconstructing the lines of descent. However, the splitting into distinct stable phenotypes always happens much earlier than this, and the shorter run therefore does not impact these findings. In Figure 4 we show the lines of descent in a simulation that after 5,000 updates resulted in four stable phenotypes (Table 5).

**Table 5:** Four stable phenotypes after 5,000 updates

Blue	0	0	0.3	0	0.4	0	0	0	0.2
Red	0	0.3	0.3	0	0.3	0	0.1	0	0.1
Green	0	0.1	0	0.4	0	0	0.4	0	0
Cyan	0.2	0.1	0	0	0	0.4	0	0.4	0.1

Colors refer to the lineages in Figure 4.

Tracking these four ecotypes revealed that the first two bifurcations happened very early on, while the last took place at update 2,916 (Table 6). The first mutational event is a double mutation that increases affinity for the seventh and ninth resources at update 15. This is an extremely advantageous mutation, with a selection coefficient of *s*=11.271. Not surprisingly, this organism swept to fixation, leading to fast depletion of those resources. As a consequence those resources conferred considerably less fitness than when the organism was rare. The split between the red and cyan lineages in Figure 4 was first marked by an increase in affinity in the red lineage for resource three at update 40. However, at this point the red lineage could have outcompeted the cyan lineage, but an increase in affinity for resource two at update 67 ensured that the cyan lineage had an advantage over the red lineage in utilizing this resource. Only with both of these mutations can the two phenotypes coexist through negative frequency-dependent selection. Similarly, the split between the green and cyan lineages at update 134 does not result in two separate ecotypes until at least one mutation later in the green lineage at update 225. The split between the red and the blue lineages also required at least two mutations at updates 2,916 and 2,999 to establish these two diverging phenotypes as ecotypes.

**Table 6:** **Complete mutational history of all four lines of descent in Table **5

**Update**	**Phenotype**									**s**
	**Blue lineage**									
1	0.1	0	0	0	0	0	0	0	0	
15	0.1	0	0	0	0	0	0.1	0	0.1	11.271
40	0.1	0	0.1	0	0	0	0.1	0	0.1	0.30965
89	0.1	0	0.1	0	0.1	0	0	0	0.1	0.039084
237	0	0	0.1	0	0.1	0	0	0	0.1	0.019431
612	0	0	0.2	0	0.1	0	0	0	0.1	0.29279
1124	0	0.1	0.2	0	0.2	0	0	0	0.1	0.27836
1393	0	0.2	0.2	0	0.2	0	0	0	0.1	0.10041
2121	0	0.2	0.2	0	0.3	0	0	0	0.1	0.10848
2658	0	0.2	0.2	0	0.3	0	0	0	0.2	0.050309
2911	0	0.2	0.3	0	0.3	0	0	0	0.2	0.077457
2916	0	0.1	0.3	0	0.3	0	0	0	0.2	-0.052489
2999	0	0.1	0.3	0.1	0.3	0	0	0	0.2	0.030072
3196	0	0	0.3	0	0.3	0	0	0	0.2	-0.044106
3249	0	0	0.3	0.1	0.3	0	0	0	0.2	0.037581
3541	0	0	0.3	0.1	0.4	0	0	0	0.2	0.067862
3802	0	0	0.3	0.2	0.4	0	0	0	0.2	0.06681
4243	0	0	0.3	0.1	0.4	0	0	0	0.2	-0.067928
4878	0	0	0.3	0	0.4	0	0	0	0.2	-0.027721
	**Red lineage**									
1	0.1	0	0	0	0	0	0	0	0	
15	0.1	0	0	0	0	0	0.1	0	0.1	11.271
40	0.1	0	0.1	0	0	0	0.1	0	0.1	0.30965
89	0.1	0	0.1	0	0.1	0	0	0	0.1	0.039084
237	0	0	0.1	0	0.1	0	0	0	0.1	0.019431
612	0	0	0.2	0	0.1	0	0	0	0.1	0.29279
1124	0	0.1	0.2	0	0.2	0	0	0	0.1	0.27836
1393	0	0.2	0.2	0	0.2	0	0	0	0.1	0.10041
2121	0	0.2	0.2	0	0.3	0	0	0	0.1	0.10848
2658	0	0.2	0.2	0	0.3	0	0	0	0.2	0.050309
2911	0	0.2	0.3	0	0.3	0	0	0	0.2	0.077457
3028	0	0.3	0.3	0	0.3	0	0	0	0.2	0.046359
3095	0	0.3	0.3	0	0.3	0	0	0	0	-0.040099
3684	0	0.3	0.3	0	0.3	0	0.1	0	0	0.036444
4850	0	0.3	0.3	0	0.3	0	0.1	0	0.1	-0.0017963
	**Green lineage**									
1	0.1	0	0	0	0	0	0	0	0	
15	0.1	0	0	0	0	0	0.1	0	0.1	11.271
67	0.1	0.1	0	0	0	0	0.1	0	0.1	0.37720
75	0.1	0.1	0	0	0	0	0.1	0.1	0.1	0.18758
225	0.1	0.1	0	0.1	0	0	0.1	0.1	0.1	0.17634
329	0.1	0.1	0	0.1	0	0	0.2	0.1	0.1	0.08945
548	0.1	0.1	0	0.1	0	0.1	0.2	0.1	0.1	0.16142
642	0	0.1	0	0.1	0	0.1	0.2	0.1	0.1	-0.0049659
800	0	0.1	0.1	0.1	0	0.1	0.2	0.1	0.1	0.094182
1060	0	0.1	0	0.1	0	0.1	0.2	0.1	0.1	-0.087319
1230	0	0.1	0	0.1	0	0.1	0.3	0.1	0.1	0.076287
1741	0	0.1	0	0.2	0	0.1	0.3	0.1	0.1	0.10556
2704	0	0.1	0	0.2	0	0.1	0.4	0.1	0.1	0.05857
2725	0	0.1	0	0.2	0	0	0.4	0.1	0.1	-0.025019
2855	0	0.1	0	0.2	0	0	0.4	0.1	0.2	0.052496
2995	0	0.1	0	0.2	0	0	0.4	0.1	0	-0.058368
3570	0	0.1	0	0.3	0	0	0.4	0.1	0	0.087326
4859	0	0.1	0	0.4	0	0	0.4	0	0	0.068163
	**Cyan lineage**									
1	0.1	0	0	0	0	0	0	0	0	
15	0.1	0	0	0	0	0	0.1	0	0.1	11.271
67	0.1	0.1	0	0	0	0	0.1	0	0.1	0.3772
75	0.1	0.1	0	0	0	0	0.1	0.1	0.1	0.18758
134	0.1	0.1	0	0	0	0	0.1	0.2	0.1	0.20596
241	0.1	0.1	0	0	0.1	0	0.1	0.2	0.1	0.13729
369	0.1	0.1	0	0	0.1	0.1	0.1	0.2	0.1	0.19802
377	0.1	0.1	0	0	0.1	0.2	0.1	0.2	0.1	0.17998
655	0.1	0	0	0	0.1	0.2	0.1	0.2	0.1	-0.042485
1776	0.1	0	0	0	0.1	0.2	0.1	0.3	0.1	0.0667
1868	0.1	0	0	0	0.1	0.2	0.1	0.4	0.1	0.055831
2112	0.2	0	0	0	0.1	0.2	0.1	0.4	0.1	0.076066
2115	0.2	0	0	0	0.1	0.3	0.1	0.4	0.1	0.04995
2283	0.2	0	0	0	0.1	0.4	0.1	0.4	0.1	0.039907
2669	0.2	0	0	0	0.1	0.4	0	0.4	0.1	-0.00022735
4124	0.2	0	0	0	0	0.4	0	0.4	0.1	-0.0067946
4545	0.2	0.1	0	0	0	0.4	0	0.4	0.1	0.013724

The selection coefficient is *s* ***=*** *W* ***′/*** *W-1*, where *W* ***′*** is the fitness of the offspring, and *W* is the fitness of the parent.

### Variable population size

To examine the effects of assuming a constant population size we ran the simulations with a variable population size as a control. In this instance of the model, all individuals are given a chance to reproduce equal to their fitness. Because fitness given by eq. (1) is always between zero and one, we can use fitness as this probability. At low resource influx, the population occasionally goes extinct when it cannot diversify from the initial specialist phenotype fast enough to gain high fitness. We therefore started the variable population size simulations with a homogeneous population of generalists where all affinities are equal to 0.1. Note that whether one starts with generalists or specialists makes no difference for the degree of specialization (Table 4).

The variable population size simulation is very sensitive to the balance between the resource influx and decay. If the decay is too high or the influx too low, the population quickly goes extinct. On the other hand, if the influx is very high, or decay is low, the population quickly grows to sizes beyond 3,000, which makes the simulation unwieldy. For these reasons we were unable to run the variable population size simulations with resource influx lower than *λ*=2·10^-4^ and higher than 4·10^-3^. Within this range the population becomes stable when the rate at which individuals are removed (1*%*) is equal to the average fitness. This range of influx values results in population sizes ranging from *N*=100 to 2,520 (Table 7).

**Table 7:** Allowing the population to vary in size does not affect the degree of specialization

**log** ***λ***	***μ***	***σ***_**1**_	***σ***_**2**_	**〈** ***n***_***t***_**〉**	***B***	***N***
-3	0.01	1	0.1	2.90±0.55	0.62	720 constant
-3	0.01	1	0.1	2.95±0.51	0.63	721 variable
-3	0.05	1	0.1	4.45±0.76	0.40	750 constant
-3	0.05	1	0.1	4.20±0.52	0.40	755 variable
-2.4	0.05	10	1	9.00±0.00	0.11	2520 constant
-2.4	0.05	10	1	8.75±0.55	0.12	2520 variable

The number of ecotypes stays the same between constant and variable population size simulations for each parameter set. The population size given is the mean of twenty runs, which varies very little; values range between 695 and 799 for the mean of 755, 696 and 784 for the mean of 721, and from 2469 to 2613 for the mean of 2520.

Results for variable population sizes are comparable to those with a constant population size. A larger population size does increase the number of ecotypes (Table 3), but there is no significant difference between the constant and variable population size simulations as long as the stable population size in the variable treatment is the same as the constant population size.

## Discussion

Trade-offs are ubiquitous in nature as species wrestle with the benefits and drawbacks of trait value optimization. In the absence of trade-offs the populations would evolve to become a generalist “superspecies” [28], and ultimately a few species would dominate, with extinction, geography and stochastic processes being the only motors of diversity. Antagonistic pleiotropy was previously found to be the primary cause of resource specialization and niche breadth reduction in the digital life software platform Avida [29], in which self-replicating computer programs evolve. However, trade-offs cannot be modified in Avida to investigate this effect quantitatively. We therefore studied sympatric, asexual populations using an individual-based model with discrete traits where fitness is an explicit function of resource consumption, and added trade-offs to this model by adding a simple cost-function to the Monod equation. We found that populations of asexual organisms in sympatry fragment into specialist ecotypes via adaptive radiation, with the degree of specialization determined largely by the severity of trade-offs. Diversification consistently occurs when resources vary enough that selection can distinguish between different phenotypes, and negative frequency-dependent selection can prevent rare phenotypes from being outcompeted. The action of negative frequency-dependent selection is contingent on the presence of trade-offs to give specialists a fitness advantage over generalists.

The origin and maintenance of generalists is only observed at high influx, while specialists dominate at low influx. High resource influx results in a population of generalists because in this case there is little competition for resources. In that case the resource abundance is always so high that both the resource maximum and minimum, *Nλ*/*c*_0_ and *Nλ*, respectively, give *r*_*k*_/(*r*_*k*_+*γ*_*k*_) close to one (eq. 1). When this happens, the amount of resource available makes no difference for selection, and there is thus no benefit to losing affinity in order to reduce cost, because all resources are abundant enough that using them at all is advantageous. On the other hand, when the resource influx is low, then the resource abundance is always low compared to the half-saturation, *γ*_*k*_, and any change in resource abundance affects fitness nearly linearly. Thus, at low resource influx selection can easily differentiate between an organism that only uses a scarce resource from one that uses an abundant one. We can also state this by saying that specialization does not happen when the population is always well-fed, but it occurs readily when the population is on the brink of starvation. Only when resource abundance is generally low does the environment induce the population to diversify. This is reminiscent of the *paradox of enrichment* in ecology, in which an ecosystem may collapse when resources are very abundant [30].

The sustained degree of specialization at low influx is unlike experiments in Avida [26], where specialization was observed only at intermediate resource influx, and not at very low or very high influx. In Avida the reduction in degree of specialization at low influx is likely due to the fact that organisms can reproduce at a low rate even in the absence of resources. This makes it difficult for selection to distinguish between different phenotypes, and thus impairs the action of negative frequency-dependent selection to sustain specialization. In our study the effect of having a constant population size is that only relative fitness matters, and relative fitness is only minimally affected by lowering the influx. A model with a forced constant population size therefore does not capture the fact that low absolute fitness, caused by fewer available resources, should decrease the total reproductive output of the population, and thereby decrease the population size. When we relaxed this assumption the population became very sensitive to the exact rate of resource flowing into the system. Indeed, we found that the range of influx that can support a population is quite narrow. Low resource influx quickly leads to extinction, while high influx lead to a population explosion that quickly becomes difficult to handle computationally. For small population sizes selection is unable to differentiate between individuals with different phenotypes. As a consequence the population drifts, disabling negative frequency-dependent selection, which is otherwise the motor of specialization. Apart from this difference, when the influx gives rise to stable populations, the variable population size implementation gives results comparable to those simulation in which the population size is fixed.

Trait-lethal mutations were introduced because it is generally easier to destroy than create function [31-33]. Many pathways for metabolizing various nutrient sources in bacteria are linear and have no redundancies (e.g., maltose, arabinose, and idonate in *E. coli*[34-36]), and loss of any one gene in these chains will disrupt the trait (genetic robustness will dampen this effect [37]). Trait-lethals make it difficult to increase the affinity of all traits at the same time, because they reduce the affinities to zero. Phenotypes that have high affinity for many resources therefore become rare in the population, and yet among the organisms on the line of descent very few instances of trait-lethals that decrease affinity by more than 0.1 are observed (the most extreme case observed was from 0.2 to 0). The mutational scheme with trait-lethals implemented in this model enhances the drive of trade-offs towards specialists, and we therefore hypothesize that specialization and niche breadth reduction are amplified by this mechanism in natural haploid asexual organisms.

The route by which adaptive radiation occurs is very informative about the evolution of specialization. By reconstructing the complete evolutionary history of each of the surviving ecotypes in a single simulation, we can track the exact mutational changes on the lines of descent. The changes in phenotype over time show that when the first split of one ecotype into two occurred (i.e., at the moment of incipient speciation), it did so by mutational changes to the phenotype that in hindsight would not have been enough to sustain the split without subsequent mutations. If the first change that separated the two lineages had be the only difference between them, one of them would have outcompeted the other in a zero-mutation rate experiment. Only through continued phyletic evolution did the first mutations lead to specialization and an increase in the number of ecotypes. This mirrors the gradual emergence of a stable polymorphism in *E. coli*, where three separate mutations in regulatory genes were needed to produce frequency-dependent effects [38].

## Conclusion

In the model presented here it is clear that trade-offs are needed for specialization and adaptive radiation to occur. Sex and spatial heterogeneity can drive specialization and diversification (e.g., [39,40]), but here we see that they are not necessary components as long as trade-offs in resource utilization are present. Specialization happens when resources are scarce, but only when the benefit of utilizing resources is constrained by trade-offs. Trait-lethal mutations that disrupt trait function enhance the drive towards specialization in synergy with trade-offs.

We have outlined an example of gradual, phyletic evolution wherein the first steps toward speciation do not in themselves complete speciation. Different lineages are only sustained as ecotypes by negative frequency-dependent selection after continued specialization on different resources. Mechanistic insight into incipient speciation could be gained by quantifying the effects of zero-mutation rate experiments shortly after such events occur. Investigating these details in multiple simulation runs would also make it possible to speak to the generality of these findings.

Trade-offs are often created on a genetic level by antagonistic pleiotropy. Since there is no genetics in the Monod model, we have instead modeled trade-offs by an explicit mathematical function. Trade-offs could be modeled within a framework that has an explicit genetic basis that includes epistasis and pleiotropy, such as the NK model [41]. In such a model genetic constraints can emerge naturally from the interaction between genetic elements, much as in the emergence of Dobzhansky-Muller incompatibilities [42], which will make it possible to study a wider range of genotype-phenotype map effects on speciation and the creation of biological diversity.

## Methods

We simulate evolution by subjecting organisms to reproduction, mutation, and selection. Each organism *i* has a phenotype consisting of as many traits as there are resources, *R*. Each trait is a *resource affinityc*_*i* *k*_, which describes the efficiency with which an organism utilizes resource *k*. All resources are perfectly substitutable. The population size *N* is kept constant by randomly removing 1*%* of the population every computational update (equivalent to about 100 updates per generation), and replacing those organisms by randomly choosing which survivors reproduce with a probability based on their relative fitness. This replacement scheme is equivalent to a death-birth Moran process with multiple deaths/births per update (see e.g., [41,43]). Offspring inherit the phenotype of the parent, but every trait value *c*_*i* *k*_ has a chance *μ* of mutating, resulting either in a decrease or an increase in resource affinity. Resource affinities vary between 0 (minimum utilization) and 1 (maximum utilization). 30*%* of mutations change a trait value by either increasing or decreasing resource affinity by 0.1 with equal probability. 70*%* of mutations are *trait-lethals*, which have the effect of setting the resource affinity to zero. This is meant to stimulate diversification by disrupting the function of utilizing one distinct resource. Once a trait is affected by a trait-lethal mutation, a subsequent mutation may again increase its value by 0.1.

Resource competition among organisms is modeled in a way similar to Michaelis-Menten kinetics and models based on the Monod equation [7,20,44], which describes fitness of each possible phenotype. Individual fitness is a function of affinity, *c*_*i* *k*_, to each resource, *k*, the resource abundance, *r*_*k*_, and of how costly it is to have a nonzero affinity (eq. 1). The half-saturation, *γ*_*k*_, is the resource abundance at which fitness from resource *k* is half of its maximal value:

(1) $$\begin{array}{l} F_{i}=\frac{1}{1+D_{i}}\underset{k}{\sum }\frac{c_{\text{ik}}r_{k}}{\left(r_{k}+\gamma_{k}\right)}. \end{array}$$

The term 1/(1+*D*_*i*_) is a sum over resource affinities that simulates trade-offs by assigning a cost to positive (*σ*_1_) and in particular multiple (*σ*_2_) resource affinities:

(2) $$\begin{array}{l} D_{i}=\underset{k}{\sum }(\sigma_{1}c_{\text{ik}}+\sigma_{2}\xi (c_{\text{ik}})). \end{array}$$

Here *σ*_1_ specifies the cost of having a high affinity for any particular resource *k*, in terms of diminishing returns for affinity on additional resources. The reasoning behind this model of diminishing returns is simple: enzymes do not act on their own. Rather, their effects are linked through their substrates and products [45,46], and increasing the affinity for one resource is likely (but not guaranteed [47]) to limit affinity for another. Even when metabolic pathways are completely modular (so that there are limited or absent diminishing returns) there can be a constant one-time cost of switching the metabolic pathway to a different resource. The parameter *σ*_2_ specifies this cost without differentiating between high and low affinity: in (2), *ξ*(*c*_*i* *k*_) is 0 for *c*_*i* *k*_=0 and 1 otherwise, distinguishing only between zero and non-zero affinity. As a typical example of such a cost, when the methylotroph *Methylobacterium extorquens* switches to growth on methanol (from its more usual succinate substrate), it needs to reset the metabolic pathway even though the two pathways are very modular [48]). As this resetting takes a few hours, it results in a fixed cost of switching. Similarly, when the evolved citrate-utilizing type of *E. coli* switches from glucose to citrate utilization it experiences a costly lag [49], and evolutionary optimization of the pathway to a large extent reduces this lag, thus, reducing the cost of switching.

Each resource is replenished every update at a constant influx per individual *λ*_*k*_, decays at a rate *c*_0_ proportional to the amount of available resource, and is consumed by the organisms at a rate equal to the total fitness in the population derived from that resource, $\underset{i}{\sum }F_{\text{ik}}$, where *F*_*i* *k*_ is the fitness derived from the *k*th resource by the *i*th individual (eq. 3).

(3) $$\begin{array}{l} \frac{{\text{dr}}_{k}}{\text{dt}}=N\lambda_{k}-c_{\;\;0}r_{k}-\underset{i}{\sum }F_{\text{ik}}. \end{array}$$

Resource abundance is at a maximum when there is no consumption, in which case we solve eq. (3) for $\frac{{\text{dr}}_{k}}{\text{dt}}=0$ with $\underset{i}{\sum }F_{\text{ik}}=0$ and obtain *r*=*Nλ*/*c*_0_. When a resource is fully consumed every update before it can decay (*c*_0_=0), the resource abundance is just equal to the amount of resources that flows into the system, *r*=*Nλ*.

### Degree of specialization

With a mutation rate of *μ*=0.05, phenotype space is fairly well explored, consistently resulting in hundreds of unique phenotypes (i.e., unique combinations of resource affinities) within a few hundred updates. To find the number of *ecotypes* in the population after 40,000 updates of reproduction, we identify stable phenotypes by performing a *zero-mutation rate experiment*. With the mutation rate set to to zero no new phenotypes are generated, and existing phenotypes begin to outcompete each other. We continue simulations until the remaining phenotypes cannot outcompete each other (no more than 80,000 updates). If this steady-state of the population was in doubt, we performed *invasion experiments* to establish the stability of the population. Invasion experiments were performed by setting one of the phenotypes to a low frequency and checking if this phenotype had the ability to invade when rare. No inconsistencies were found between the two methods.

We quantify the *degree of specialization* by the number of ecotypes *n*_*t*_ and the population *niche breadth* (e.g., [50]), calculated as the fraction of nonzero resource affinities in the population:

(4) $$\begin{array}{l} B=\underset{i,k}{\sum }\xi (c_{\text{ik}})/\mathrm{NR.} \end{array}$$

*B* equals 1/*R* when every individual utilizes exactly one resource, and 1 when every individual uses all resources. A population of generalists is thus characterized by a high niche breadth, while resource specialization results in low niche breadth. This definition is close to that used in investigations of niche breadth reduction in Avida [29], where it is calculated as the proportion of organisms performing each function summed over all functions, rather than taking the mean over all resources, as we do here.

### Initial conditions

We start simulations with a homogeneous population comprised of specialists that have one nonzero affinity *c*_*i*1_=0.1 and the rest equal to zero, or with generalists that have affinity 0.1 for all resources. Throughout we use *R*=9 resources, *c*_0_=0.1, and *γ*_*k*_=100 for all resources. We start with all resource abundances either at a minimum equal to the influx, *Nλ*, or at the maximum given by eq. (3) without consumption, *Nλ*/*c*_0_, but we emphasize that distinction makes no difference to the evolution of the population, because resource abundances adjust based on consumption before the population has enough time to change how they use resources.

## Availability of supporting data

The data set supporting the results of this article is available in the Dryad repository, doi:10.5061/dryad.6n660 http://dx.doi.org/10.5061/dryad.6n660[51].

## Competing interests

The authors declare that they have no competing interests.

## Authors’ contributions

BØ and RL wrote the code and carried out the simulations. BØ performed the data analysis. BØ and CA conceived of the study and participated in its design and coordination, and drafted the manuscript. All authors read and approved the final manuscript.
